# Identification of SOFT syndrome caused by a pathogenic homozygous splicing variant of *POC1A*: a case report

**DOI:** 10.1186/s12920-021-01055-1

**Published:** 2021-08-21

**Authors:** Guoqiang Li, Guoying Chang, Chen Wang, Tingting Yu, Niu Li, Xiaodong Huang, Xiumin Wang, Jian Wang, Jiwen Wang, Ruen Yao

**Affiliations:** 1 Department of Medical Genetics and Molecular Diagnostic Laboratory, Shanghai Children’s Medical Center, School of Medicine, Shanghai Jiao Tong University, Shanghai, 200127 People’s Republic of China; 2 Department of Endocrine and Metabolism, Shanghai Children’s Medical Center, School of Medicine, Shanghai Jiao Tong University, Shanghai, 200127 People’s Republic of China; 3 Department of Neurology, Shanghai Children’s Medical Center, School of Medicine, Shanghai Jiao Tong University, Shanghai, 200127 People’s Republic of China

**Keywords:** SOFT syndrome, POC1A, Splicing variant, Short stature, Case report

## Abstract

**Background:**

Pathogenic variants in *POC1A* led to SOFT syndrome and variant POC1A-related (vPOC1A) syndrome. SOFT syndrome is a rare primordial dwarfism condition characterized by short stature, onychodysplasia, facial dysmorphism and hypotrichosis.The main clinical differences between SOFT and vPOC1A syndrome include dyslipidemia with insulin resistance and acanthosis nigricans. To our knowledge, this is the first report of a SOFT syndrome patient diagnosed with a homozygous splicing variant, which could help to extend our understanding of the genotypic and phenotypic information of the disease.

**Case presentation:**

We reported a seven-year-old boy with SOFT syndrome. The patient presented symmetrical short stature and facial features, including prominent forehead, inverted triangular face, epicanthal fold, small teeth and enlarged ears. Laboratory tests displayed mild insulin resistance. Whole-exome sequencing (WES) led to the identification of a homozygous splicing variant (c.981+1G>A) in *POC1A* gene of the patient, which was inherited from his heterozygous parents confirmed by Sanger sequencing. Further transcriptional experiments of the splicing variant revealed aberrant percentage of exon 9 skipping transcripts.

**Conclusions:**

This is the firstly reported case of a SOFT syndrome patient with a novel homozygous splicing variant and detailed delineation of the aberrant transcript in proband and carrier of the variant in Chinese. Our study enriched mutational spectrum of *POC1A* which could help in further genetic diagnosis and counselling of SOFT syndrome patients.

**Supplementary Information:**

The online version contains supplementary material available at 10.1186/s12920-021-01055-1.

## Background

SOFT syndrome (OMIM 614813) is a rare genetic disorder, which is the abbreviation of Short stature, Onychodysplasia, Facial dysmorphism and hypoTrichosis, initially termed by Sarig et al. in 2012 [1]. SOFT syndrome is caused by *POC1A* gene mutation and is inherited in an autosomal recessive manner. There are three reviewed mRNA isoforms for *POC1A* gene, one of which lacks exon 10. *POC1A* gene has pleiotropic effects, because truncating variants locating in exon 10 could cause variant POC1A-related (vPOC1A) syndrome, whose main clinical features included an extreme dyslipidemia with insulin resistance, acanthosis nigricans and short stature. To date, only 15 families with molecularly confirmed variants in *POC1A* gene have been reported [1–12]. *POC1A* gene encodes the POC1 centriolar protein A, which is one of the two proteome of centriole 1 (POC1) proteins in humans. The encoded protein consists of an N-terminal WD40 domain and a C-terminal coiled coil domain, named the Poc1 motif, and is involved in centriole duplication and length control [13]. Here, we report the first Chinese patient of SOFT syndrome harboring a homozygous splicing variant of *POC1A* gene.

## Case presentation

The proband was a seven-year-old boy. He was referred to the endocrinology clinic of Shanghai Children’s Medical Center (SCMC) for the chief complaint of short stature and facial dysmorphism. He was the third child of non-consanguineous parents of the Han nationality. His parents had an induced labour for absence of one kidney for fetus. His parents were physically healthy without relevant family history. The height of his father was 172 cm and mother 160 cm (Fig. 2a).

**Fig. 1:**
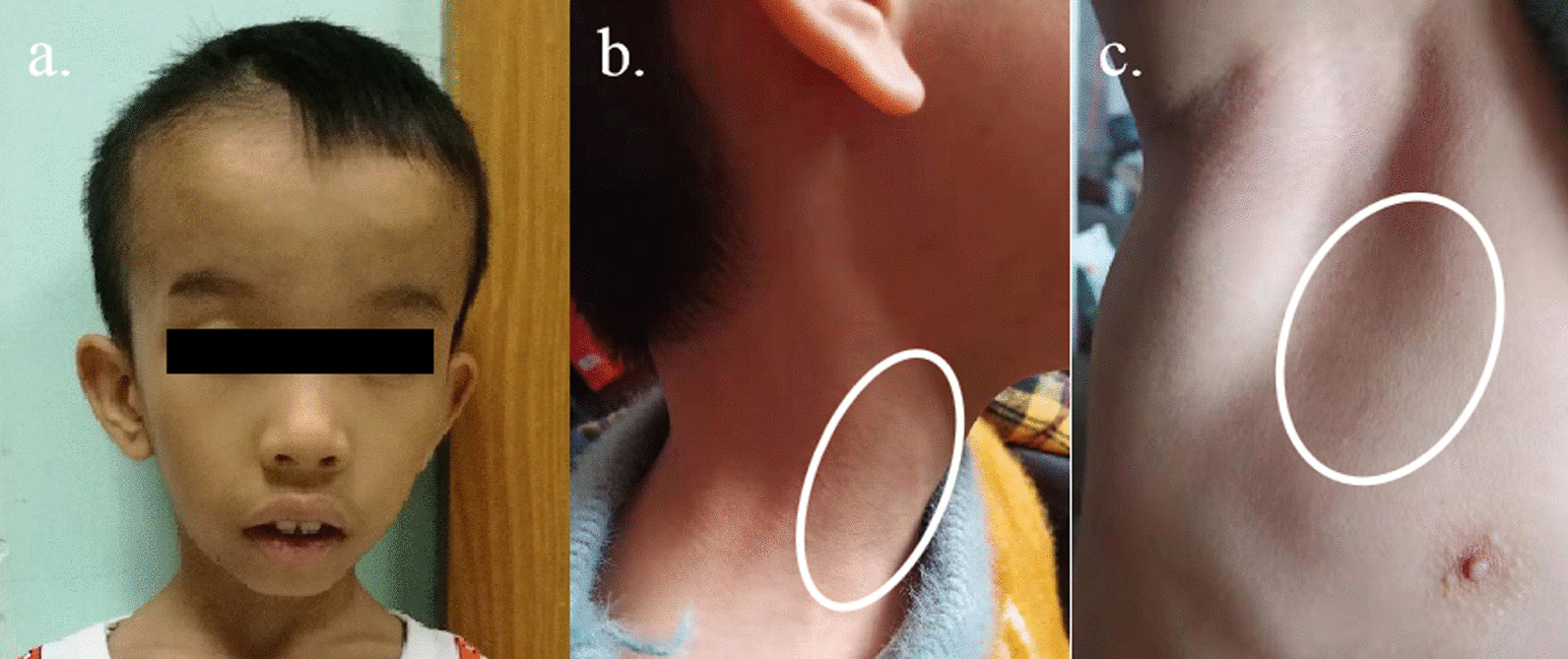
The phenotype of the patient. **a** Facial dysmorphism including prominent forehead, inverted triangular face, epicanthal fold, small teeth and enlarged ears. **b**, **c** Mild acanthosis nigricans inneck and axillary fossa, the acanthosis nigricans was indicated by a circle

The child was delivered by normal labor at full term gestation. During pregnancy, prenatal examination indicated that he had short legs and arms. His birth weight was 2300 g [− 2.6 SD] and length at birth was 46.8 cm [− 2 SD]. When ten months old, he was taken to the local hospital due to growth retardation and small appetite, and a diagnosis of thyroid dysgenesis was made. Then, he took the sodium levothyroxine for 1 year, but stopped taking it because of no efficacy. Mild speech delay and motor developmental delay were described by his parents. The height he gained was less than 1 cm in the past year. His intelligence was normal. Upon physical examination, his height was 90 cm (− 6.3 SD), weight 10.3 kg (− 3.9 SD), and his head circumference was 48 cm (< − 2 SD). He presented facial dysmorphism including prominent forehead, inverted triangular face, epicanthal fold, small teeth and enlarged ears (Fig. 1a). And mild acanthosis nigricans occurred in his neck and axillary fossa (Fig. 1b, c). His vision and hearing was normal.

The laboratory results showed mild insulin resistance. Because of his compliance, we examined his blood glucose and insulin via a simple oral glucose tolerance test. The results showed that the fasting glucose value was 5.05 mmol/L (reference 4.1–5.9 mmol/L), and the fasting insulin value was 90.28 pmol/L (reference 12-84 pmol/L). And two hours after having a meal, the level of glucose was 5.29 mmol/L (reference 4.1–5.9 mmol/L), and the insulin was 433.99 pmol/L (reference 12–84 pmol/L). The level of HbA1c was 5.6% (reference 4.0–6.0%). The level of insulin like growth factor 1 (IGF1) and insulin like growth factor binding protein 3 (IGFBP3) was 190.0 ng/ml (− 0.5 SD) and 3.84ug/ml (− 1.5 SD), respectively. The Vitamin D was 20.0 ng/ml (reference > 30.0 ng/ml). Laboratory investigations showed levels within the reference ranges including free triiodothyronine (FT3), free tetraiodothyronine (FT4) and thyroid stimulating hormone (TSH). Moreover, the liver and kidney function, blood cholesterol and blood triglycerides were also normal. The echocardiography was normal but the electrocardiograph indicated a prolongation of the QT interval when tachycardia. The X-ray indicated a bone age of 3.5-year-old. And the ultrasound of heart and abdomen was normal.

In order to detect disease-causing mutations, genomic DNA was extracted from peripheral blood samples of patient and his parents using the Gentra Puregene Blood Kit (Qiagen, Hilden, Germany) according to the manufacturer’s protocol. Whole exome capture was performed with Agilent SureSelect V6 enrichment capture kit (Agilent Technologies, Inc., Woburn, MA, U.S.) according to the manufacturer’s instructions. The captured library was then sequenced on an Illumina HiSeq 2500 System (Illumina, Inc., San Diego, CA, U.S.). Original sequencing data were assessed using FastQC (version 0.11.2) for quality control. The Burrows Wheeler Alignment tool (BWA) v0.2.10 was employed for sequencing data alignment to the Human Reference Genome (NCBI build 37, hg 19). Single nucleotide variants and small indels were identified by GATK. All variants were saved in VCF format and uploaded to the Ingenuity Variant Analysis (Ingenuity Systems, Redwood City, CA, USA) and TGex (Translational Genomics Expert) platform for biological analysis and interpretation. Variants detected by next-generation sequencing were confirmed by Sanger sequencing in the patient and his parents. The primers designed for exon 9 were as follows: forward 5′-CTCAGAACTTTGGGCATGGC-3′ and reverse 5′-CTAAACCTCCTCCCTCACCG-3′. Transcriptional experiments were performed to evaluate the splicing effects of the variant. The cDNA products were amplified with primer P1, 5′-CTCGTATTGTGAGCATGGCG-3′, which was located in the exon6; and primer P2, 5′-GATTCCTGCTCCCCTGATCA-3′, which was located in exon11. The polymerase chain reaction (PCR) products were further cloned into the pMD 19 T vector (Takara Biotechnology [Dalian] Co., Ltd.). The polymerase chain reaction was carried out in a C1000™ Thermal Cycler PCR instrument (Bio-Rad Laboratories). The resulting DNA was sequenced using an ABI3730XL sequencer (Thermo Fisher Scientific) with the forward and reverse primers. The sequence data were analyzed using Mutation Surveyor^®^ software version 4.0.4 (SoftGenetics, LLC).

**Fig. 2:**
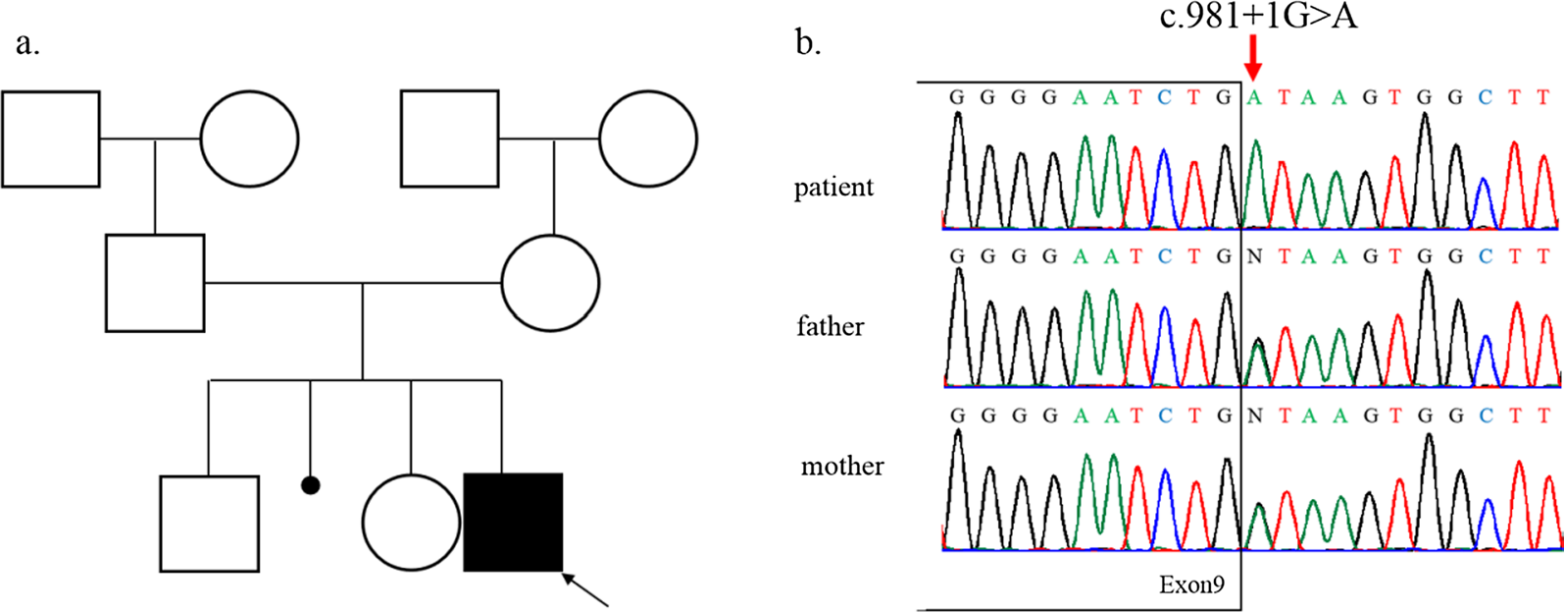
**a** The pedigree of the patient. The proband was indicated by an *arrow*. **b** Identification of a splicing variant in the *POC1A* gene. Sequences show a homozygous splicing variant (c.981+1G>A in intron 9) in the patient. The patient’s father and mother were both carriers. Red arrows, mutant bases

Whole-exome sequencing revealed a novel homozygous splicing variant, c.981+1G>A, in intron 9 of the *POC1A* gene (NM_015426.5) in the patient. Sanger sequencing confirmed the variant and heterozygous status in his father and mother (Fig. 2b). The variant detected in the family was not included in control population database (gnomAD) and our local control cohorts database.

Reverse transcription polymerase chain reaction (RT-PCR) performed on RNA extracted from the patient, parents and control subject, revealed the existence of aberrant transcripts percentage derived from the splicing variant. Besides the shorter isoform of *POC1A* lacking exon 10 which had been proved to existed in different tissues, a significant altered percentage of mutant isoform lacking exon 9 and both exon 9&10 had been discovered between the proband, the parents and control sample (Table 1, Additional file 1: Data 1). The exon 9 and exon 9&10 skipping transcripts both leading to short protein product with in-frame deletion of 33 and 81 amino acids respectively.

**Table 1:** isoforms of POC1A transcript detected in control sample and the pedigree samples

	Full-length isoform	10-isoform	9-isoform	9- and 10- isoform
Control	62/74 (83.78%)	12/74 (16.22%)	0/74 (0.00%)	0/74 (0.00%)
Carrier (father)	29/76 (38.16%)	5/76 (6.58%)	34/76 (44.74%)	8/76 (10.53%)
Carrier (mother)	27/76 (35.53%)	3/76 (3.95%)	37/76 (48.68%)	9/76 (11.84%)
Proband	0/80 (0.00%)	0/80 (0.00%)	69/80 (86.25%)	11/80 (13.75%)

## Discussion and conclusions

SOFT syndrome is characterized by short stature, facial dysmorphism with hypotrichosis and facial dysmorphism. Pathogenic variants in *POC1A* gene can lead to SOFT syndrome. The growth retardation can be detected on prenatal ultrasound [1, 2]. Relative macrocephaly is present in childhood but the head circumference becomes smaller with advancing age [1, 2]. Skeletal anomalies like clinodactyly, brachydactyly and hypoplastic distal phalanges have been reported. Recognizable facial dysmorphism includes elongated triangular face, prominent nose and abnormal ears [6]. In addition, affected patients have a high-pitched voice [9]. The patients usually have normal psychomotor development and intelligence.

**Fig. 3:**
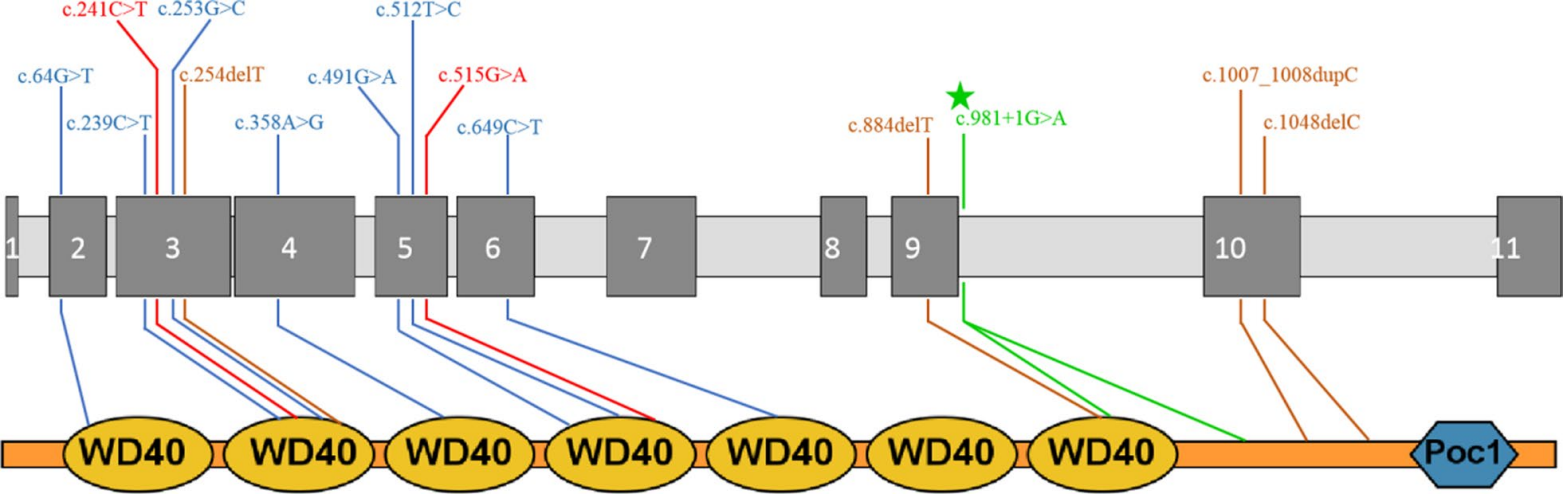
Positions of variants within the *POC1A* gene (NM_015426.5). Missense mutations are in blue, nonsense mutations in red and frameshift mutations in brown. Variants in our study are in green

*POC1A* encodes the POC1 centriolar protein A, which plays a role in centrosome-mediated cell mitosis control via mitotic spindle organization and cilia formation. Formation of supernumerary centrosomes and multipolar spindle were observed in fibroblast cells derived from the affected individuals [1, 3]. Therefore, SOFT syndrome could be classified as a type of ciliopathy. To our best knowledge, only 13 variants of *POC1A* gene have been reported until now, including 7 missense mutations, 2 nonsense mutations and 4 frameshift mutations [1–12] (Fig. 3).

Giorgio E et al. suggested that the *POC1A* gene was a gene with pleiotropic effects, and variants affecting exon 10 only could cause variant POC1A-related (vPOC1A) syndrome instead of typical SOFT syndrome, whose main clinical features included an extreme dyslipidemia with insulin resistance and acanthosis nigricans, because variants restricted to exon 10 of the *POC1A* gene affected only two of the three mRNA isoforms, leaving a functional isoform [7]. To date, three vPOC1A syndrome individuals had been reported [4, 7, 12]. The splicing variant detected in our patient has been proven to cause exon 9 skipping which are necessary in all isoforms and affect WD40 domain integrity. The WD40 domain is necessary to localize the protein to centrioles, and defective WD40 domain will inhibit the proper protein localization. The parents, heterozygous carriers of the variant, were also significantly affected, producing only about 40% normal transcripts of *POC1A*. These findings provided evidences for possible pathogenic mechanism of dosage effect of variants in *POC1A* gene.

In current study, our patient’s phenotypes corresponded with major characteristics of SOFT syndrome, and present with an extra diagnosis of thyroid dysgenesis and mild insulin resistance, which was different from previous reported cases with SOFT and vPOC1A syndrome. We supposed that the reason was that he was at prepubertal age, when severe insulin resistance was yet to be clinically expressed [7]. Further evaluation on his metabolism is needed in follow-up visit from puberty onwards to explore the existence of severe insulin resistance, which is a significant feature for vPOC1A syndrome.

In summary, we reported the first patient with SOFT syndrome harboring a homozygous splicing variant of *POC1A* which leads to aberrant transcripts lacking exon 9 which possibly affected WD40 domain structure of the protein. The patient presented with typical SOFT syndrome features including primordial dwarfism, characteristic facial dysmorphism and delayed bone age, as well as mild insulin resistance. Identification of novel types of *POC1A* mutation in patients may provide appropriate management and correct genetic counseling to those patients and their families.

## Supplementary Information

**Additional file 1.** Data 1 contains detail presentation of normal and aberrant transcripts of the proband, his parents and normal control.

## Data Availability

The novel variant revealed during the study has been submitted to ClinVar repository with the accession number SCV001769404 (https://www.ncbi.nlm.nih.gov/clinvar/variation/1192285/). The datasets generated during the current study are available in the NCBI Sequence Read Archive Database (Accession Number: PRJNA753550) (https://www.ncbi.nlm.nih.gov/bioproject/PRJNA753550). The relevant datasets links used in this study were as follows: Human Reference Genome (GRCh37/hg19) (http://hgdownload.soe.ucsc.edu/goldenPath/hg19/bigZips/hg19.fa.gz), gnomAD (https://gnomad.broadinstitute.org), 1000 Genomes project (http://www.1000genomes.org/), dbSNP (http://www.ncbi.nlm.nih.gov/snp), ClinVar (https://www.ncbi.nlm.nih.gov/clinvar/), and OMIM (http://omim.org).

## Abbreviations

WES: Whole-exome sequencing
PCR: Polymerase chain reaction
RT-PCR: Reverse transcription polymerase chain reaction
SD: Standard deviation
