# Supplementary material for: Identification of SOFT syndrome caused by a pathogenic homozygous splicing variant of POC1A: a case report
Source: BMC Med Genomics. 2021 Aug 21;14:207. doi: 10.1186/s12920-021-01055-1 (PMC8379828; doi:10.1186/s12920-021-01055-1)

control

exon9

exon8


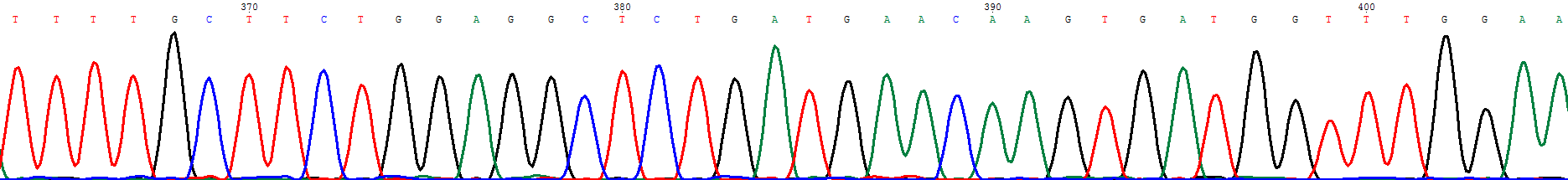


exon9

exon10


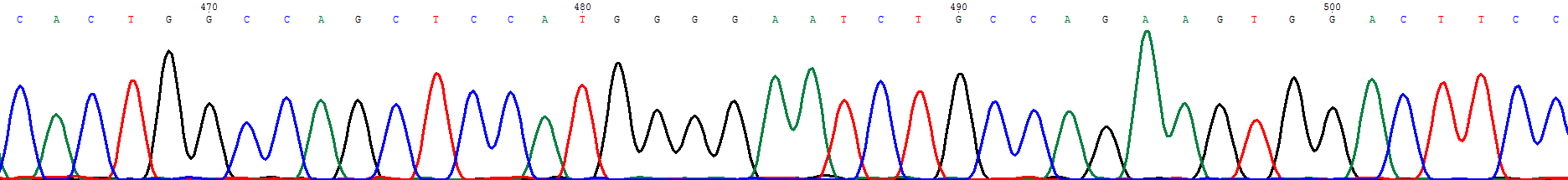


exon11

exon9


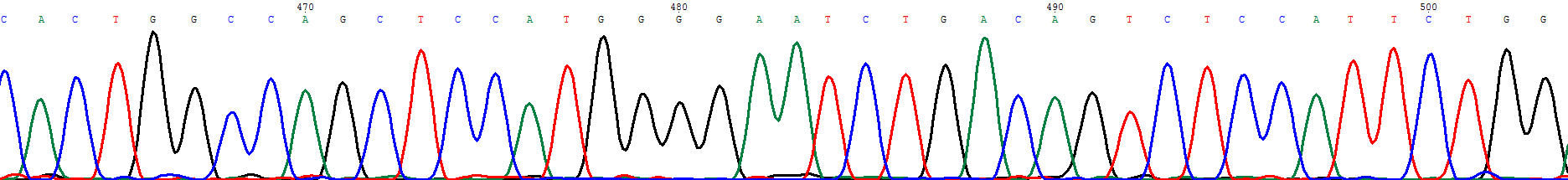


proband

exon9

exon11


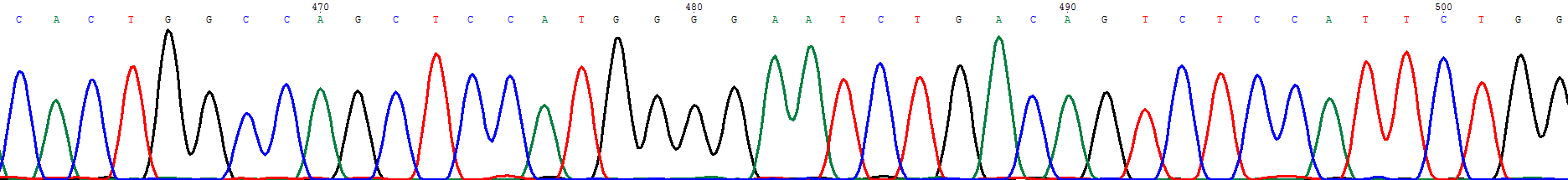


exon8

exon11


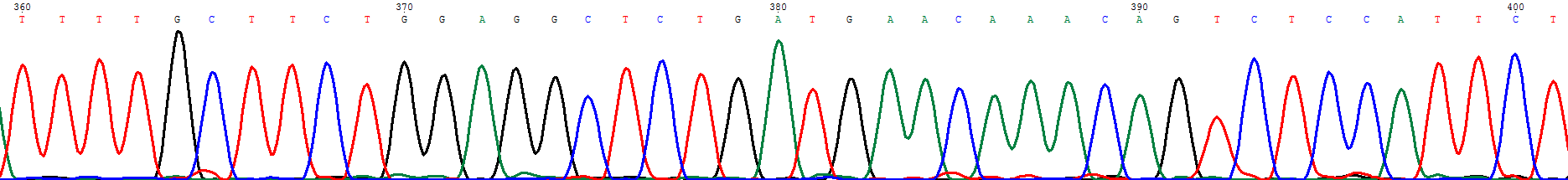


father

exon10

exon9


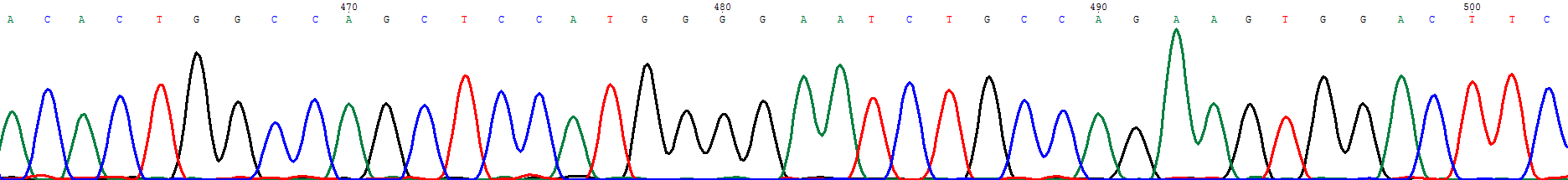


exon11

exon9


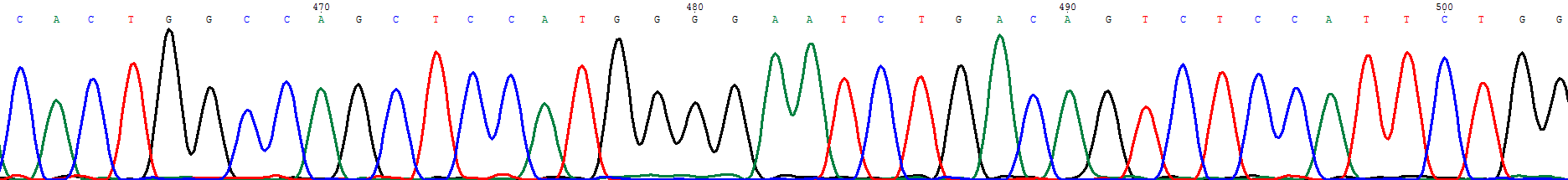


exon9

exon8


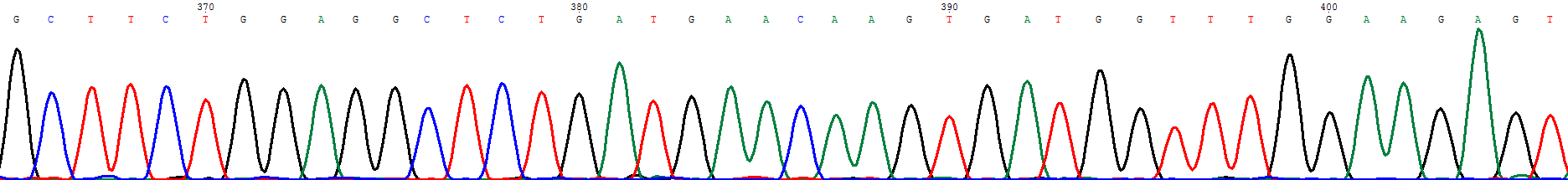


exon10

exon8


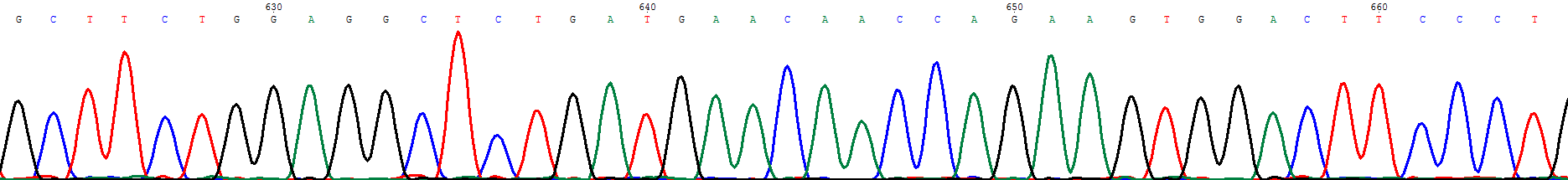


exon11

exon8


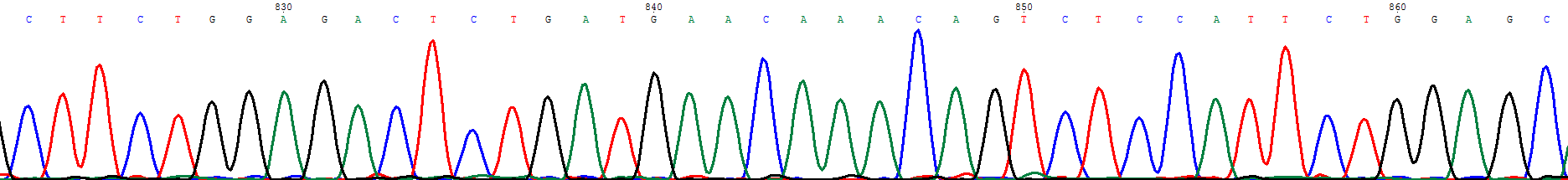


mother

exon10

exon9


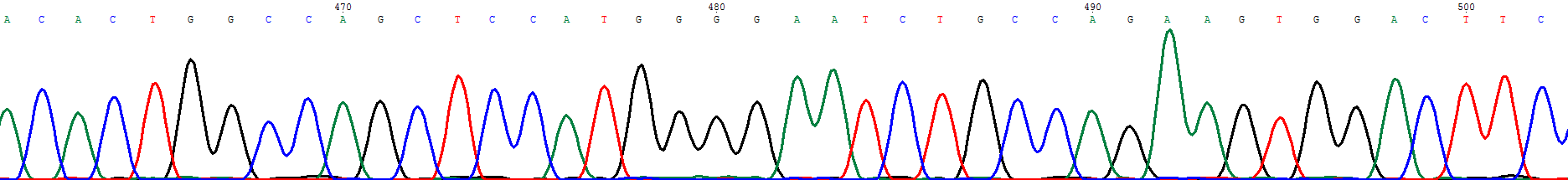


exon11

exon9


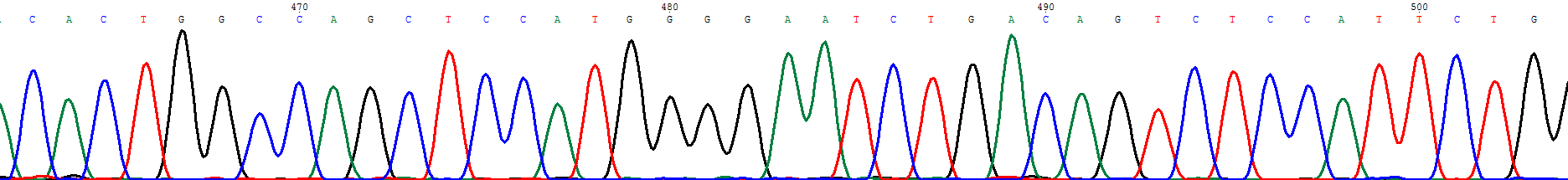


exon9

exon8


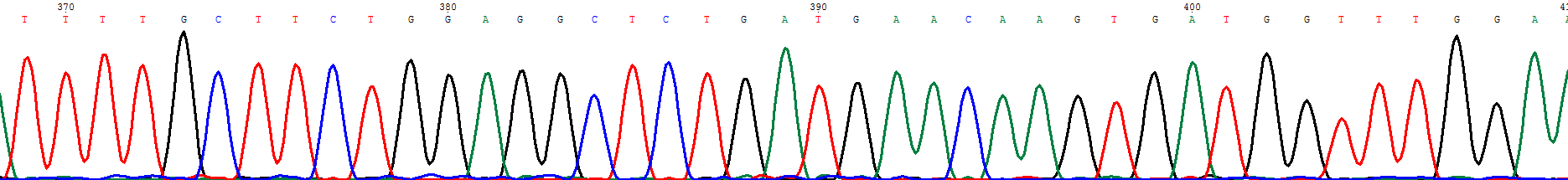


exon10

exon8


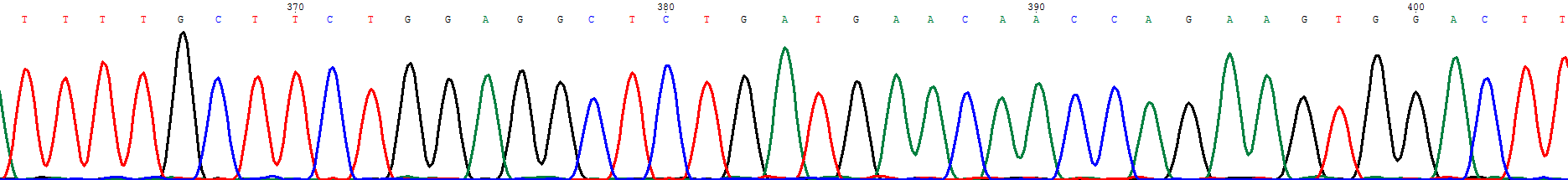


exon11

exon8


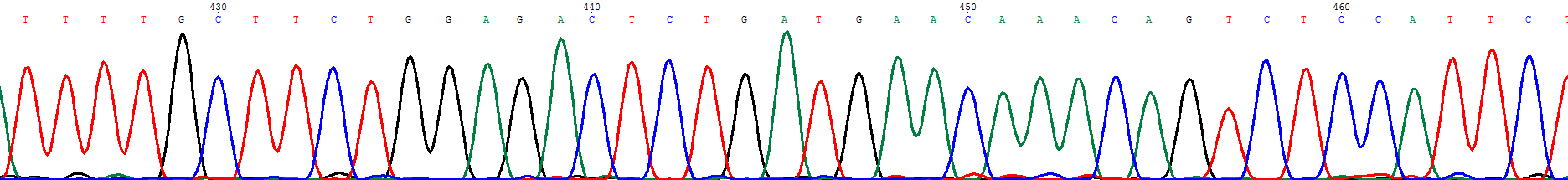

Supplement: Supplementary file 1 — Additional file 1. Data 1 contains detail presentation of normal and aberrant transcripts of the proband, his parents and normal control. [file 12920_2021_1055_MOESM1_ESM.docx]
